# Three-year adherence to secondary prevention and vascular risk control after ischemic stroke

**DOI:** 10.1093/esj/23969873251329210

**Published:** 2026-01-01

**Authors:** Erlend Fagerli, Hanne Ellekjær, Olav Spigset, Ingvild Saltvedt, Mari Nordbø Gynnild

**Affiliations:** Department of Circulation and Medical Imaging, NTNU – Norwegian University of Science and Technology, Trondheim, Norway; Department of Neuromedicine and Movement Science, NTNU – Norwegian University of Science and Technology, Trondheim, Norway; Stroke Unit, Clinic of Medicine, St. Olavs Hospital, Trondheim University Hospital, Trondheim, Norway; Department of Clinical Pharmacology, St. Olavs Hospital, Trondheim University Hospital, Trondheim, Norway; Department of Clinical and Molecular Medicine, NTNU – Norwegian University of Science and Technology, Trondheim, Norway; Department of Neuromedicine and Movement Science, NTNU – Norwegian University of Science and Technology, Trondheim, Norway; Department of Geriatric Medicine, St. Olavs Hospital, Trondheim University Hospital, Trondheim, Norway; Department of Circulation and Medical Imaging, NTNU – Norwegian University of Science and Technology, Trondheim, Norway; Stroke Unit, Clinic of Medicine, St. Olavs Hospital, Trondheim University Hospital, Trondheim, Norway; Clinic of Cardiology, St. Olavs Hospital, Trondheim University Hospital, Trondheim, Norway

**Keywords:** Secondary prevention, ischemic stroke, stroke, medication adherence, vascular risk factors, blood pressure

## Abstract

**Introduction:**

Long-term adherence to secondary prevention after ischemic stroke remains unclear. This study aimed to evaluate medication adherence, attainment of vascular treatment targets, and clinical characteristics that influence target achievement 3 years post-stroke.

**Patients and methods:**

We included 665 home-dwelling ischemic stroke patients from the Norwegian Cognitive Impairment After Stroke study, admitted between May 2015 and March 2017 (*n* = 431 were followed for 3 years). Medication adherence was assessed using the 4-item Morisky Medication Adherence Scale, medication persistence, and guideline-based treatment targets: blood pressure (BP) < 140/90 mmHg, LDL cholesterol (LDL-C) < 2.0 mmol/L, and hemoglobin A1c (HbA1c) ⩽ 53 mmol/mol.

**Results:**

At discharge, prescription rates were 97% for antithrombotics, 67% for antihypertensives, 88% for lipid-lowering drugs (LLD), and 10% for antidiabetics. Three years later, persistence rates were 97%, 91%, 83%, and 94%, respectively, with 73% reporting high medication adherence. Target achievement rates were 42% for BP, 47% for LDL-C, and 75% for HbA1c among diabetic patients. Younger age was associated with better BP control (OR 0.974 per year, 95% CI 0.957–0.992). Women had poorer LDL-C control (OR 0.55, 95% CI 0.33–0.91). More LLD (OR 1.25, 95% CI 1.14–1.37) and higher comorbidity (OR 1.26, 95% CI 1.10–1.44) were associated with improved LDL-C control.

**Conclusion:**

Control of risk factors remained unsatisfactory 3 years after ischemic stroke, despite relatively high persistence and adherence rates. Improved focus on implementing optimal secondary prevention for Norwegian stroke patients is necessary.

## Graphical Abstract

**Graphical abstract:**
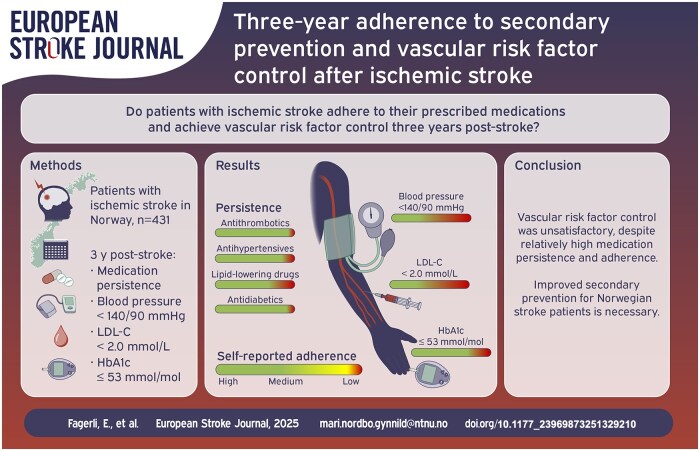

## Introduction

Stroke patients are at high risk of recurrent vascular events.^1,2^ Effective implementation of secondary prevention strategies can offer substantial health and socioeconomic benefits, and modeling studies have shown that approximately 80% of recurrent events could potentially be avoided by optimal secondary prevention.^3^ However, studies have consistently shown that secondary prevention following stroke, myocardial infarction and other vascular events remains suboptimal.^4–6^ A previous study from our group has shown that less than half reach the treatment targets for blood pressure (BP) and low-density lipoprotein cholesterol (LDL-C) after 3 and 18 months post-ischemic stroke.^7^ Additionally, several studies show that medication adherence decreases over time for patients with chronic diseases.^8,9^ Recent studies assessing the quality of long-term secondary prevention post-stroke, especially with follow-up periods extending beyond 18 months, are limited. Therefore, this study aimed to assess medication persistence and adherence, vascular risk factor control and clinical characteristics influencing target achievement 3 years after ischemic stroke.

## Patients and methods

### Study population

**Figure 1.**
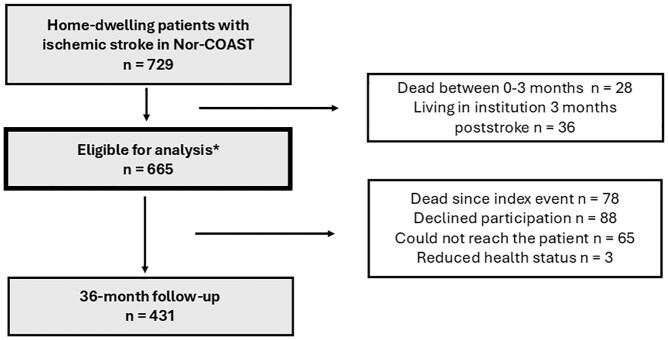
Flowchart for inclusion and exclusion of the participants. *Patients included in previous work.^7^

This substudy is part of the Norwegian Cognitive Impairment After Stroke (Nor-COAST) study, a prospective multicenter cohort study involving patients from five Norwegian stroke units.^10^ Participants with acute ischemic stroke were recruited between May 2015 and March 2017 and followed after 3 months, 18 months, and 3 years through questionnaires, clinical interviews, exams, and blood samples. Patients unable to attend in person were assessed by phone. In total, 665 home-dwelling patients were eligible for analysis, while 78 patients died before the 3-year follow-up and 431 patients attended the 3-year follow-up, Figure 1. Ethical approval was granted (REC 2017/1462), and informed consent was obtained.

### Baseline assessments

Demographics, comorbidity, vascular risk factors and medications at the index stay were retrieved from medical records, patients/proxies and clinical examination.^10^ Medication lists were collected from the patients’ discharge summary.

### Outcome assessments

At 3-years, participants underwent blood tests, BP measurements, physical and cognitive tests, and interviews with patients/next of kin. A self-report questionnaire covered medication adherence, smoking, lifestyle and post-stroke function.

#### Medication persistence and adherence

Medication persistence was defined as the continued use of preventive medications prescribed at discharge. Trained professionals collected data through interviews with participants or proxies, consulting general practitioners, home care services, or the Norwegian summary care record (Kjernejournalen). Drug classes included: antithrombotics (Anatomical Therapeutic Chemical (ATC) Classification System code B01A), antihypertensives (C03A, C07, C08, C09A/B, C09C/D, and C02A/C/D), lipid-lowering drugs (LLD; C10), and antidiabetics (A10). Medication adherence was assessed by the 4-item Morisky Medication Adherence Scale (MMAS-4),^11–13^ which is a general medication-taking behavior scale. Each item in the MMAS-4 has a dichotomous response option. The sum creates a total score ranging from 0 to 4 with 4 indicating high adherence, 2–3 medium and 0–1 low adherence (Supplemental Methods).*

#### Treatment target achievements

Treatment targets followed Norwegian guidelines at the time of the study.^12^ BP was measured three times by trained professionals at the 3-year visit with 1-min intervals, with the average of the last two measurements used. Venous blood samples assessed HbA1c and LDL-C. BP control was defined as BP < 140/90 mmHg, HbA1c ⩽ 53 mmol/mol and smoking cessation. LDL-C control was defined as LDL-C < 2.0 mmol/L, as most physicians were probably treating toward this target at the time of the study, however, LDL-C ⩽ 1.8 mmol/L was also assessed.^13^ Changes in BP and LDL-C were also evaluated during follow-up.

#### Clinical characteristics influencing target achievement for patients using pharmacotherapy

Potential factors influencing target achievement was chosen a priori and included: age, sex, education (years), living alone, frailty, cognitive function, number of medications used, medication adherence assessed by MMAS-4, Charlson comorbidity index (CCI), and psychological distress, measured using the hospital anxiety and depression scale (HADS).

### Statistical analysis

We used descriptive statistics for baseline characteristics, medication adherence and target achievement, reporting means (standard deviations (SD)) for continuous variables, and percentages for categorical variables. Target achievement was assessed overall and among patients using relevant drugs, including LDL-C targets for patients using high-intensity statins (⩾40 mg atorvastatin or equivalent). Statin equivalency was calculated using WHO-defined daily doses (DDDs). Logistic regression identified characteristics associated with target achievement for patients using pharmacotherapy, and linear regression with BP and LDL-C as continuous outcomes. We first did available case analyses for patients attending the 3-year visit (*n* = 431). However, because patients lost-to-follow-up most likely have different target achievements and adherence to medications, we also used mixed model logistic and linear regression which is less biased than available case analysis,^14^ including all participants which potentially could have attended the 3-year follow-up (*n* = 587). Proportions reaching targets at each time-point were calculated by odds converted to probability (*p*) by *p* = odds/(1 + odds) for all participants and separately for those using relevant pharmacotherapy. We also assessed absolute changes in LDL-C and BP during follow-up. Statistical significance was set at *p* < 0.05, with cautious interpretation for *p*-values between 0.01 and 0.05. Analyses were performed using SPSS 29 and Stata 18.0.

## Results

### Baseline characteristics

Table 1 presents characteristics at the index stay and 3-year follow-up. Prescription rates for secondary preventive medications were 97% (*n* = 420) for antithrombotics, 67% (*n* = 288) for antihypertensives, 88% (*n* = 381) for LLD, and 10% (*n* = 42) for antidiabetics. Table S1 shows that patients lost to follow-up were older, less educated, less physically active, and had more comorbidities, worse functional status, and poorer cognitive function prestroke.

**Table 1.** Baseline and 3-year characteristics (*n* = 431).

Age, years	70.6 (11.2)
Sex, female	185 (43%)
Education, years	12.5 (3.7)
Living alone	125 (29%)
Home care	21 (5%)
Charlson Comorbidity Index	3.6 (1.9)
Cognitive impairment	31/426 (7%)
Frail	36 (8%)
Hypertension	224 (52%)
Atrial fibrillation	89 (21%)
Diabetes mellitus	67 (16%)
Previous stroke or TIA	80 (19%)
Previous ischemic heart disease	71 (17%)
Chronic kidney disease	64/429 (15%)
Smoking	86 (20%)
Body mass index, kg/m^2^	26.3 (4.0)
Physically inactive	318 (74%)
NIHSS at admission	2 (1–4)
NIHSS at discharge	1 (0–2)
Independent functional status at discharge	309/429 (72%)
Number of medications at discharge	4.9 (2.5)
TOAST classification	
Large artery atherosclerosis	36/419 (9%)
Cardioembolism	101/419 (24%)
Small artery occlusion	94/419 (22%)
Stroke of other determined cause	14/419 (3%)
Stroke of undetermined cause	174/419 (42%)
Clinical features at 3 years post-stroke	
Independent functional status	257/424 (61%)
Number of medications in use	4.2 (3.3)
NIHSS	0 (0–1)
Cognitive impairment	123/419 (29%)

TIA: transient ischemic attack; NIHSS: National Institutes of Health Stroke Scale.

Values are mean (standard deviation or interquartile range) or *n* (%). Definitions are described in Supplemental Methods.

### Medication persistence and adherence at 3 years

**Figure 2.**
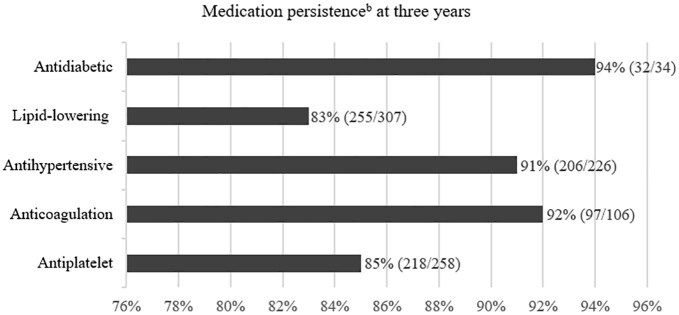
Persistence to secondary preventive medications at 3 years (*n* = 349^a^). Values are % (*n/N*). ^a^Participants with available follow-up data on medications. ^b^Persistence to prescribed medication within the class at hospital discharge.

Follow-up data on medication persistence were available for 81% (*n* = 349), with 97%, 91%, 83%, and 94% persistence rates for antithrombotics, antihypertensives, LLD, and antidiabetics, respectively (Figure 2).

Of 345 patients completing the MMAS-4,73% (*n* = 251) reported high adherence, 25% (*n* = 87) medium, and 2% (*n* = 7) low adherence, respectively.

Among 418 patients with information on medication administration, 80% (*n* = 336) managed medications independently, while 20% received assistance from next of kin (*n* = 19) or healthcare personnel (*n* = 63). Among those with persistence data, 84% (*n* = 291) administered medications independently.

### Treatment target achievement at 3 years

Table 2 shows proportions achieving treatment targets via mixed model logistic regression. Figure S1 shows for available cases.

**Table 2.** Proportions achieving vascular risk factor control at 3 years.

	All patients			Patients prescribed pharmacotherapy^e^		
Risk factor control at 3 years	*n* ^f^	Probability	95% CI (%)	*n* ^f^	Probability	95% CI (%)
Blood pressure control^a^	576	41.6%	34.2–49.4	455	43.4%	35.1–52.1
LDL cholesterol control^b^	584	43.7%	33.6–54.4	537	46.9%	36.3–57.9
Glycemic control^c + d^	103	85.2%	50.6–96.9	87	69.4%	30–92.3

LDL: low density lipoprotein.

Based on mixed model logistic regression with time point as categorical covariate and patient as random effect.

^a^Blood pressure (BP) < 140/90 mmHg.

^b^LDL cholesterol < 2.0 mmol/L.

^c^HbA1c ⩽ 53 mmol/mol.

^d^For patients with diabetes mellitus (DM), defined as using blood glucose lowering drugs at admission or discharge or HbA1c ⩾ 48 mmol/mol at admission or self-report of diet-regulated DM.

^e^Prescribed pharmacotherapy at discharge and/or anytime during the 3 years of follow-up, for blood pressure control; on antihypertensives, for LDL control; on lipid lowering drugs, for glycemic control; on antidiabetic medication.

^f^Living participants at 3 years with at least one measurement at baseline, 3 months, 18 months, or 3 years.

Mean BP was 140/82 mmHg (SD 20/12) with 41.6% achieving the target; among antihypertensive users, 43.4% reached the target. Non-achievers had a mean BP of 154/88 mmHg (SD 14/10), using a mean number of 1.7 (SD 0.9) antihypertensive drugs, whereas 54% used only one drug. In total, 12.0% achieved BP < 130/80 mmHg estimated by mixed model logistic regression (21% (61/286) for available cases).

Mean LDL-C was 2.3 mmol/L (SD 0.9) with 43.7% achieving the target < 2.0 mmol/L. For those using LLD, 46.9% met the target. Among LLD users not reaching the target, 52% (*n* = 39) were on high-intensity statins. Among 271 LLD users, 7% (*n* = 20) used ezetimibe, while none used proprotein convertase subtilisin/kexin type 9 (PCSK9) inhibitors. In total, 31.1% reached LDL-C < 1.8 mmol/L.

For diabetes patients, mean HbA1c was 48 mmol/mol (SD 9.5), with 85.2% meeting the target. Among antidiabetic users, 69.4% reached the target. Of patients smoking at baseline, 85% had quit after 3 years. Overall, 25% (51/208) achieved all treatment targets. At 3 years, 27% (75/275) had minimum of 75 min high-intensity exercise or 150 min moderate-intensity exercise per week. Mean BMI was 26.8 (SD 5.0).

### Factors influencing vascular risk factor control

Table 3 shows characteristics influencing the control of vascular risk factors, with age, sex and education-adjusted results in Table S2. Advanced age was associated with reduced odds for BP target achievement (OR 0.974/year, 95% CI 0.957–0.992, *p* = 0.004). Mean age was lower among those who met the BP target (66.8 years vs 70.9 years, *p* = 0.002). Advanced age was associated with increased odds for LDL-C control (OR 1.03 per year, 95% CI 1.00–1.05, *p* = 0.030). Females had reduced odds for LDL-C control (OR 0.55, 95% CI 0.33–0.91, *p* = 0.021) compared with men. Comorbidity and numbers of drugs used were associated with increased LDL-C control (OR 1.26, 95% CI 1.10–1.44, *p* = 0.001) and OR 1.25, 95% CI 1.14–1.37, *p* ⩽ 0.001), respectively). Number of medications in use were also associated with increased BP control (OR 1.08, 95% CI 1.01–1.15, *p* = 0.022). However, no significant associations were found with education, adherence, cognitive function, frailty, or HADS. LDL-C and systolic BP as continuous outcome variables (Table S3) aligned with dichotomous outcomes. Table S4 shows characteristics for patients with stable, decreasing and increasing LDL-C and systolic BP levels from 3 months to 3 years for those prescribed pharmacotherapy. Patients with increasing LDL-C levels (*n* = 169) had lower statin dose, and patients with increasing BP levels (*n* = 90), were older and had a lower number of antihypertensive drugs. Table S5 presents target achievement stratified by stroke etiology.

**Table 3.** Mixed model logistic regression with vascular risk factor control as dependent variable, for participants prescribed pharmacotherapy.

Predictor	Blood pressure control^a^			LDL cholesterol control^b^		
	*n* *	OR (95% CI)	*p*	n*	OR (95% CI)	*p*
Age, years	455	0.974 (0.957–0.992)	0.004	537	1.03 (1.00–1.05)	0.030
Female	455	0.98 (0.67–1.41)	0.894	537	0.55 (0.33–0.91)	0.021
Education, years	455	0.98 (0.93–1.03)	0.406	537	1.00 (0.93–1.07)	0.913
Living alone	455	0.81 (0.56–1.18)	0.273	537	1.20 (0.74–1.98)	0.457
Frailty^c^	455	0.98 (0.82–1.18)	0.850	537	1.13 (0.88–1.45)	0.345
Cognitive function^d^	453	1.01 (0.87–1.18)	0.901	535	1.09 (0.89–1.33)	0.399
Number of drugs	455	1.08 (1.01–1.15)	0.022	537	1.25 (1.14–1.37)	<0.001
Self-reported medication adherence^e^	391	1.00 (0.75–1.32)	0.977	426	0.94 (0.69–1.28)	0.705
Comorbidity^f^	455	0.95 (0.86–1.05)	0.336	537	1.26 (1.10–1.44)	0.001
HADS^g^	387	1.00 (0.96–1.04)	0.992	426	1.00 (0.96–1.05)	0.978

OR: odds ratio; CI: confidence interval.

For both models results are adjusted for time point as categorical covariate and patient as random effect.

^*^Living participants at 3 years (n = 587) with at least one measurement at baseline, 3 months, 18 months, or 3 years.

^a^Blood pressure < 140/90 mmHg.

^b^LDL cholesterol < 2.0 mmol/L.

^c^Fried criteria 0–5, with 0, and as reference corresponding to robust 5 to frail.

^d^Measured by Global Deterioration Scale 1–7, with 1 as reference (normal cognitive function).

^e^Morisky Medication Adherence Scale 4, with range 0–4, and 4 as reference, corresponding to high adherence.

^f^Charlson Comorbidity Index with 0 as reference.

^g^Hospital Anxiety and Depression Scale 0 – 42, with 0 as reference with increasing scores indication increasing burden.

## Discussion

### Main findings

We observed high persistence and self-reported adherence to secondary preventive medications 3 years after ischemic stroke. Despite this, less than half of the participants met BP and LDL-C targets. Younger patients had better BP control, while females had poorer LDL-C control. Additionally, a higher number of LLD and greater comorbidity were associated with improved LDL-C control. Although 98% of patients reported medium to high medication adherence, only a quarter successfully controlled all key risk factors.

### Persistence and self-reported adherence rates

Our study showed higher persistence rates than similar studies.^15,16^ However, antiplatelet therapy persistence declined to 85%, likely due to transitions from antiplatelets to anticoagulants, and persistence rate for LLD declined to 83%, consistent with other studies.^17^ Interestingly, a longitudinal study surveying patients 1 year post-ischemic stroke or transient ischemic attack (TIA) identified healthcare provider cessation, rather than patient self-discontinuation, as the primary cause of non-persistence.^18^ Non-persistence may result from clinical decisions related to quality of life, polypharmacy, and side effects; though, our study did not capture these aspects.^19^

Self-reported medication adherence in our study was high compared to others, with a meta-analysis finding an overall 64% high adherence rate to secondary preventive medications post-stroke.^16^ Our high rates may reflect healthier patients attending the 3-year follow-up, potentially causing selection bias, as well as the self-reporting nature of MMAS-4.

### Target achievement and clinical characteristics of influence

Our findings align with other studies showing unsatisfactory BP and LDL-C target achievement.^4,5^ A recent Swedish study also reported suboptimal risk factor control and a stagnation of BP and LDL-C control 1 year after TIA.^9^ We found lower BP control compared to the Tromsø Study^4^ and Swedish TIA patients,^9^ but slightly higher than a Norwegian study post-coronary events and EUROASPIRE (stroke module).^5,6,20^ Interestingly, the EUROASPIRE surveys showed improvements over time, unlike our stable measurements over 3 years.^20^ However, these studies are not directly comparable due to differences in study duration, patient populations and healthcare access.

The LDL-C and glycemic control were higher compared to similar studies. Using an LDL-C cut-off of < 1.8 mmol/L, control rates would align with EUROASPIRE,^20^ be higher than the Tromsø Study,^4^ but lower than in Norwegian coronary patients.^6^ However, comparisons are challenging due to differing follow-up durations, target cut-offs and disease focus. Glycemic control was poorer in patients on antidiabetics, consistent with prior follow-ups.^7^

Several factors may explain why treatment targets are not being met.^21,22^ High BP, LDL-C and glucose levels are often asymptomatic, increasing the risk of nonadherence and clinical inertia.^23^ Women had poorer LDL-C control, consistent with previous studies showing lower adherence and doses of LLD in women.^24,25^ Notably, 52% of patients not meeting LDL-C targets were on high-intensity statins. Most patients using LLD had stable LDL-C levels, despite being above target thresholds (Table S4). We demonstrated that using more LLD improved LDL-C control, consistent with existing research.^26–28^ Additionally, most patients not reaching the BP targets used only one antihypertensive agent.

BP control declined with increasing age. Hypertension is more prevalent in elderly, and discussions of BP thresholds, considering potential risks of side-effects, may explain a less intensive treatment approach. Stroke etiology might also play a role (Supplemental Table S5), as evidence has been more robust for LDL-C lowering in patients with coronary artery disease and atherosclerotic large-artery stroke, and guidelines often differentiate between stroke etiologies.^29^ Stroke is heterogenous, and it might sometimes better harmonize with the individual’s benefit, treating targets less intensively, with physicians taking a pragmatic approach when levels are close to targets.

### Strengths and limitations

A key strength of the present study is the multicenter design, prospectively and consecutively including an unselected stroke cohort, with a longitudinal follow-up and multiple measurements.^7^ Other studies might be hampered by low participation rates and retrospective and cross-sectional design.^5^ The broad inclusion criteria make our findings applicable to the average Norwegian stroke patient, although they were slightly younger than the national average.^30^ However, our study has limitations. The 3-year follow-up may have selected healthier individuals, as those lost to follow-up were older and had more comorbidity. We minimized this bias using model-based estimates.^14^ One-third of the patients had blood tests at 3 years. We lack reliable long-term BP data as ambulatory BP monitoring or multiple time-point measurements during the day. We also lacked data on side-effects, quality of life, and patients’ preferences. Lack of monitoring, limited awareness of recommended targets, and appropriate clinical inaction might be of importance; however, we lacked data on general practitioners’ perspectives, clinical visits and whether (or why not) treatment was intensified. Decisions regarding medication changes were made at the discretion of treating physicians, based on individual patient profiles and clinical judgment. Additionally, we had limited information on rehabilitation services, income and occupation, which could influence adherence. Self-reported adherence, clinical interviews and information from next of kin may have overestimated actual adherence due to recall and social desirability bias. While MMAS-4 is validated for Norwegian use, it is not specific to stroke patients^11^ or secondary prevention, and may not accurately reflect adherence to individual medications. High adherence does neither necessarily indicate correct medication use (i.e. correct time of day, appropriate dosage). Finally, medications alone are insufficient for comprehensive secondary prevention, and lifestyle interventions may have contributed to the observed outcomes.

### Clinical implications

Although adherence and persistence were relatively high, only half of the patients achieved BP and LDL-C targets. This pattern, observed across Europe, also applies to Norway where health resource allocation and government-covered medications potentially enhance adherence. Our cohort included mostly mild strokes, and those with severe strokes may face greater barriers to optimal risk factor control. These findings highlight challenges beyond patient-related adherence barriers, such as treatment inertia, the need for individualized care, and the importance of long-term follow-up. Regular assessment of treatment targets and adherence is crucial. Many patients not meeting LDL-C goals were on high-intensity statins, suggesting a need for adjunctive therapies like ezetimibe or PCSK9 inhibitors. Similarly, most patients not reaching BP targets were on a single antihypertensive agent. Although stroke is a heterogeneous condition, early combination therapy, dose adjustments alongside lifestyle interventions and improved primary-specialist collaboration, may optimize vascular risk management. Given the challenges identified, future research should assess the impact of lifestyle modifications using standardized and systematic methods. Studies should also focus on optimizing the implementation of secondary prevention strategies, including the role of shared decision-making and structured treatment intensification to improve adherence to preventive targets. Additionally, future research should aim to identify individuals who will benefit most from intensive management and closer long-term follow-up.

## Conclusion

Despite relatively high self-reported adherence and high persistence to medications prescribed at discharge, vascular risk factor control remains less than ideal 3 years after ischemic stroke. Patients with BP control were younger, and patients with LDL-C control had a higher number of LLD. Practical implementation of long-term secondary prevention for Norwegian stroke patients requires further insight.

## Supplementary Material

supplementary_files_23969873251329210

## Footnotes

MMAS-4 scale republished by permission of Adherence. © MMAS www.adherence.cc

## Trial registration

ClinicalTrials.gov Identifier: NCT02650531.
