# Supplementary material for: Three-year adherence to secondary prevention and vascular risk control after ischemic stroke
Source: Eur Stroke J. 2026 Jan 1;11(1):23969873251329210. doi: 10.1093/esj/23969873251329210 (PMC12863440; doi:10.1093/esj/23969873251329210)
Supplement: supplementary_files_23969873251329210 [file supplementary_files_23969873251329210.zip › sj-pdf-1-eso-10.1177_23969873251329210.pdf]

Supplementary material

Supplementary methods

Definitions used in the baseline table (Table 1): Charlson Comorbidity Index ranges from 0 to 37; higher scores corresponds to greater comorbidity, while 0 corresponds to no comorbidity [1]. Cognitive impairment defined as Global Deterioration Scale≥3 [2]. Frailty defined by 5-item Fried frailty criteria score ≥3 [3]. Hypertension defined as self-reported hypertension or use of antihypertensive drugs. Hypercholesterolemia defined by use of lipid-lowering drugs at admission. Atrial fibrillation (paroxysmal or permanent) based on hospital medical records or documented on electrocardiogram or telemetry during admission. Prestroke diabetes mellitus based on hospital medical records, hemoglobin A1c ≥ 48 mmol/mol, or prescribed antidiabetic drugs at admission. Prevalence of previous cerebrovascular disease and coronary heart disease were retrieved from hospital medical records. Chronic kidney disease defined as glomerular filtration rate <60 mL/min/1.73 m^2. Self-reported non-adherence to a minimum of 75 minutes high-intensity exercise or 150 minutes moderate-intensity exercise per week. Stroke severity according to National Institutes of Health Stroke Scale (NIHSS). Independent functional status defined as Modified Rankin Scale ≤2 [4-6]. TOAST; Trial of Org 10172 in Acute Stroke Treatment [7].

Statin equivalency was calculated using WHO-defined daily doses (DDDs). The formula used for this calculation was: (Dose of ”other statin”/DDD for ”other statin”) x DDD for atorvastatin). Example: For 20 mg of simvastatin, the atorvastatin equivalent dose is 20 mg divided by 30 (DDD for simvastatin) multiplied by 20 (DDD for atorvastatin) = 13.33 mg atorvastatin.

Predictors used in regression analyses included age, sex, number of education years, frailty, cognitive function, number of medications used, medication adherence assessed by Morisky Medication Adherence Scale (MMAS4)* [4-6], charlson comorbidity index (CCI) [1] and psychological distress, measured using the hospital anxiety and depression scale (HADS)[8]. MMAS-4 is a general medication-taking behavior scale. Each item in the MMAS-4 has a dichotomous response option (yes=0, no=1) and is based on the following questions: 1) Do you ever forget to take your mediations? 2) Do you ever have problems remembering to take your medications? 3) When you feel better, do you sometimes stop taking your medications? 4) Sometimes if you feel worse when you take your medications, do you stop taking them? The sum creates a total score ranging from 0 to 4 with 4 indicating high adherence, 2-3 medium and 0-1 low adherence.

*MMAS-4 scale republished by permission of Adherence. © MMAS www.adherence.cc

Table S1. Analysis of participant dropout after three years for the patients included at baseline (*n*=665)

	Total (*n*=665)	Still in follow-up (*n*=431)	Lost to follow-up (*n*=234)	P-value
Age, years	72.9 (11.5)	70.6 (11.2)	77.2 (10.8)	<0.001
Sex, female	288 (43%)	248 (43%)	103 (44%)	0.786
Education, years	12.1 (3.7)	12.5 (3.7)	11.26 (3.5)	<0.001
Married or cohabitant	415 (62%)	293 (68%)	122 (52%)	<0.001
Current or previous smoker	377 (57%)	237 (55%)	140 (60%)	0.217
BMI (kg/m^2)	26.1 (4.2)	26.3 (4.0)	25.8 (4.5)	0.174
Physically inactive[a]	51 (10%)	46 (14%)	5 (3%)	<0.001
Charlson Comorbidity Index	4.1 (2.0)	3.6 (1.9)	4.9 (2.0)	<0.001
Cognitively impaired, prestroke[b]	84 (13%)	31 (7.3%)	53 (23%)	<0.001
NIHSS, admission	2 (1-5)	2 (1-4)	3 (1-5)	0.023
Modified Rankin Scale, discharge	2 (1-3)	2 (1-3)	3 (1-4)	<0.001
Baseline systolic blood pressure (mmHg)	142 (20)	142 (20)	142 (20)	0.731
Baseline LDL cholesterol (mmol/L)	3.0 (1.1)	3.1 (1.1)	2.9 (1.0)	0.012
Baseline HbA1c (mmol/mol)[c]	57 (18)	56 (18)	57 (18)	0.603

Values are mean (SD) or median (interquartile range) for continuous variables using two-sample t-test or Mann-Whitney U test and proportions (%) for categorical variables using chi-square test. [a]Self-reported non-adherence to physical activity guidelines defined as minimum 75 minutes per week of high-intensity exercise or minimum 150 minutes per week of moderate intensity exercise. [b]Defined by a Global Deterioration Scale score of ≥3. [c]In patients with diabetes mellitus (n=130). Abbreviations: BMI; Body Mass Index, NIHSS; National Institutes of Health Stroke Scale, LDL; Low density lipoprotein, HbA1c; Hemoglobin A1c.

*MMAS-4 scale republished by permission of Adherence. © MMAS www.adherence.cc

Figure S1. Proportions achieving treatment targets at the three-year follow-up (available case analysis, *n*=431)

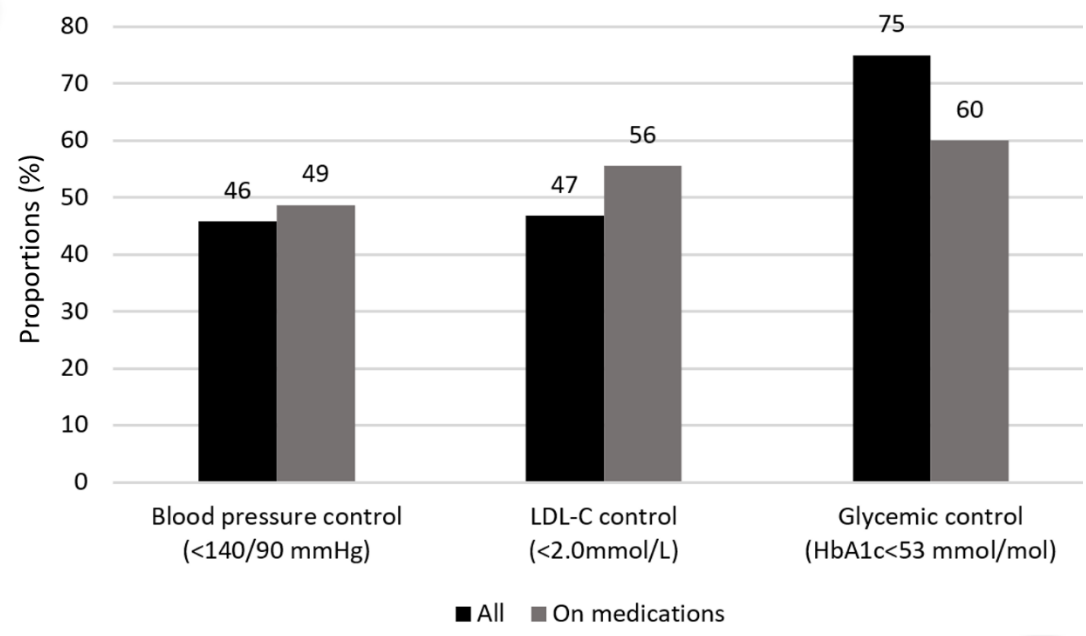

"All" corresponds to participants with data on the relevant variable (n=286 for BP control, n=222 for LDL-C control and n=24 for glycemic control). "On medications" corresponds to the use of relevant preventive medication(s) at the three-year follow-up (n=189 for BP control, n=172 for LDL-C control and n=15 for glycemic control). For BP control; on antihypertensives, for LDL-C control; on lipid-lowering drugs, for glycemic control; on antidiabetic drugs. For glycemic control we consider patients with diabetes mellitus prestroke based on hospital medical records, HbA1c ≥ 48 mmol/mol, or prescribed antidiabetic drugs at admission. Abbreviations: BP; Blood pressure, LDL-C; Low-density lipoprotein cholesterol, HbA1c; Hemoglobin A1c.

**MMAS-4 scale republished by permission of Adherence. © MMAS www.adherence.cc*

Table S2. Mixed model logistic regression with vascular risk factor control as dependent variable, for participants prescribed pharmacotherapy*, adjusted for age, sex and education

	Blood pressure control[a]			LDL cholesterol control[b]		
	n	OR (95% CI)	p	n	OR (95% CI)	p
Living alone	455	0.89 (0.61 to 1.31)	0.554	537	1.25 (0.75 to 2.10)	0.396
Frailty[d]	455	1.05 (0.86 to 1.29)	0.601	537	1.17 (0.89 to 1.55)	0.256
Cognitive function[e]	453	1.05 (0.89 to 1.24)	0.540	535	1.04 (0.85 to 1.29)	0.691
Number of drugs used	455	1.09 (1.02 to 1.16)	0.010	537	1.26 (1.15 to 1.38)	<0.001
Self-reported medication adherence[f]	391	0.99 (0.75 to 1.31)	0.961	426	0.95 (0.70 to 1.29)	0.725
Comorbidity[g]	455	1.05 (0.93 to 1.18)	0.475	537	1.23 (1.04 to 1.46)	0.016
HADS[h]	387	1.00 (0.96 to 1.03)	0.911	426	1.01 (0.96 to 1.06)	0.792

[*]Pharmacological treatment with antihypertensives for BP control and lipid lowering drugs for LDL control, for living participants at 3 years (n=587) with at least one measurement at baseline, 3 months, 18 months, or 3 years. [a]Blood pressure < 140/90 mmHg, [b]LDL cholesterol < 2.0 mmol/L. [d]Fried criteria 0-5, with 0 as reference corresponding to robust, and 5 to frail. [e]Measured by Global Deterioration Scale 1 – 7, with 1 as reference corresponding to normal cognitive function. [f]Self-reported medication adherence measured by Morisky Medication Adherence Scale 4, with range 0-4, and 4 as reference, corresponding to high adherence, 0 corresponding to low adherence. MMAS-4 scale republished by permission of Adherence. © MMAS www.adherence.cc. [g]Comorbidity measured by Chalson Comorbidity Index with 0 as reference. [h]Hospital Anxiety and Depression Scale 0 – 42, with 0 as reference with increasing scores indication increasing burden. Abbreviations: OR; Odds ratio. CI; Confidence interval.

*MMAS-4 scale republished by permission of Adherence. © MMAS www.adherence.cc

Supplementary table S3. Mixed model linear regression with systolic blood pressure and LDL cholesterol as continuous dependent variables, for participants prescribed pharmacotherapy[a].

	BP (mmHg)			LDL-C (mmol/L)		
	n	Coefficient (95% CI)	p	n	Coefficient (95% CI)	p
Age, years	455	0.34 (0.19 to 0.50)	<0.001	537	-0.01 (-0.2 to -0.003)	0.002
Sex, female	455	2.84 (-0.35 to 6.0)	0.081	537	0.21 (0.08 to 0.34)	0.001
Education, years	455	0.38 (-0.04 to 0.81)	0.077	537	0.01 (-0.01 to 0.03)	0.257
Living alone	455	2.68 (0.09 to 5.27)	0.043	537	0.03 (-0.09 to 0.16)	0.604
Frailty[b]	455	0.23 (-1.40 to 1.86)	0.783	537	-0.02 (-0.08 to 0.04)	0.517
Cognitive function[c]	453	0.77 (-0.43 to 1.96)	0.209	535	-0.02 (-0.08 to 0.03)	0.349
Number of drugs used	455	-0.72 (-1.23 to -0.20)	0.006	537	-0.07 (-0.09 to -0.05)	<0.001
Self-reported medication adherence[d]	391	-0.37 (-1.88 to 1.13)	0.628	426	0.01 (-0.04 to 0.06)	0.701
Comorbidity[e]	455	0.75 (1-0.14 to 1.65)	0.099	537	-0.09 (-0.12 to -0.05)	0.001
HADS[f]	387	0.07 (1-014 to 0.28)	0.518	426	0.003 (-0.005 to 0.01)	0.461

Results are adjusted for time point as categorical covariate and patient as random effect. [a]Pharmacological treatment with antihypertensives for BP control and lipid lowering drugs for LDL control, for living participants at 3 years (n=587) with at least one measurement at baseline, 3 months, 18 months, or 3 years. [b]Fried criteria 0-5, with 0 as reference corresponding to robust, and 5 to frail. [c]Measured by Global Deterioration Scale 1 – 7, with 1 as reference corresponding to normal cognitive function. [d]Self-reported medication adherence measured by Morisky Medication Adherence Scale 4, with range 0-4, and 4 as reference, corresponding to high adherence, 0 corresponding to low adherence. MMAS-4 scale republished by permission of Adherence. © MMAS www.adherence.cc. [e]Charlson comorbidity index [f]Hospital Anxiety and Depression Scale 0 – 42, with 0 as reference with increasing scores indication increasing burden. Abbreviations: BP; Blood pressure, LDL-C; Low density lipoprotein cholesterol, CI; Confidence interval.

*MMAS-4 scale republished by permission of Adherence. © MMAS www.adherence.cc

Table S4. Characteristics for patients within trajectories of LDL-C and systolic BP from 3 months to 3 years for those prescribed pharmacotherapy

	Stable LDL-C levels (n = 298)	Decreasing LDL-C levels (n = 120)	Increasing LDL-C levels (n=169)	Stable BP levels (n = 420)	Decreasing BP levels (n=77)	Increasing BP levels (n = 90)
Age	70.7 (11.5)	73.1 (10.4)	73.3 (12)	70.8 (12)	73.8 (9)	75.7 (10)
Sex, female	126 (42%)	71 (59%)	67 (40%)	184 (44%)	43 (56%)	37 (41%)
LDL-C at 3 months (mmol/L)	2.11	2.73	1.89	2.2 (0.8)	2.3 (0.8)	2.1 (0.6)
LDL-C at 3 years (mmol/L)	2.10	1.83	3.00	2.3 (0.9)	2.3 (0.9)	1.4 (0.9)
Systolic BP at 3 months (mmHg)	140 (20)	144 (19)	139 (19)	141 (17)	156 (23)	128 (15)
Systolic BP at 3 years (mmHg)	140 (20)	139 (21)	144 (21)	141 (18)	121 (19)	160 (18)
Change in levels (%)	0.1 (11)	-32 (10)	60 (42)	0.5 (7)	-23 (7)	25 (11)
Atorvastatin equivalent dose at 3 months (mg)	35 (23)	42 (25)	38 (22)	36 (23)	32 (23)	44 (25)
Atorvastatin equivalent dose at 18 months (mg)	30 (23)	38 (25)	27 (22)	30 (24)	30 (24)	28 (24)
Global deterioration scale at 3 years	1.8 (1.0)	2.4 (1.4)	2.1 (1.2)	1.9 (1.1)	2.3 (1.1)	2.2 (1.4)
Number of antihypertensive drugs	1.14 (1.0)	1.23 (1.1)	1.0 (1.1)	1.1 (1.0)	1.3 (1.0)	0.9 (0.9)
Optimal medication adherence	233 (78%)	90 (75%)	126 (75%)	328 (78%)	50 (65%)	71 (79%)
Charlson Comorbidity Index	3.73 (1.9)	4.04 (2.1)	3.88 (1.9)	3.6 (1.9)	4.9 (2.3)	3.8 (1.4)
HADS score	7.3 (6.2)	8.5 (6.4)	7.1 (5.9)	7.4 (6)	8.0 (6)	7.7 (6)

Values are mean (SD) or n (%) if other not specified. Stable levels for LDL-C were defined as an absolute change ≤20%, decreasing levels were defined as > 20% decrease from 3 months to 3 years and increasing levels as > 20% increase from 3 months to 3 years. For blood pressure stable levels were defined as an absolute change ≤15%. Abbreviations: BP; Blood pressure, LDL-C; Low density lipoprotein cholesterol, LLD; Lipid-lowering drugs, HADS; Hospital Anxiety and Depression Scale.

*MMAS-4 scale republished by permission of Adherence. © MMAS www.adherence.cc

Table S5. Target achievement stratified by stroke etiology according to the TOAST classification estimated by mixed model (n=569[a])										
		LDL-C < 2.0 mmol/L		LDL-C < 1.8 mmol/L			BP < 140/90 mmHg		BP < 130/80 mmHg	
	n[b]	Probability	95% CI	Probability	95% CI	n[b]	Probability	95% CI	Probability	95% CI
Large artery disease	57	27.7%	9.4 to 65.0	15.7%	3.0 to 53.1	54	32.0%	14.9 to 55.7	9.7%	2.5 to 31.1
Cardioembolic stroke	132	33.0%	15.9 to 56.0	28.8%	14.8 to 52.6	130	49.7%	32.2 to 67.2	17.0%	7.8 to 33.2
Small vessel disease	133	65.5%	41.6 to 83.4	44.7%	22.3 to 69.6	132	55.6%	39.8 to 70.3	16.0%	8.0 to 29.3
Undetermined	228	46.4%	30.7 to 62.9	36.3%	21.4 to 54.3	226	36.2%	25.6 to 48.5	11.6%	6.3 to 20.2

Based on mixed model logistic regression with time point as categorical covariate and patient as random effect. Abbreviations: TOAST; Trial of Org 10172 in Acute Stroke Treatment [7], LDL-C; low-density lipoprotein cholesterol, BP; blood pressure., CI; confidence interval. [a]n=569 of the 587 patients eligible for mixed model analyses were stratified according to the TOAST classification (Large artery disease n=57, cardioembolic stroke n =134, small vessel disease n = 133, other n = 16, undetermined source n =229). n is patients within this category with an LDL-C measurement / BP measurement at at least one time point during follow-up.

*MMAS-4 scale republished by permission of Adherence. © MMAS www.adherence.cc

References

1. Charlson ME, Pompei P, Ales KL and MacKenzie CR. A new method of classifying prognostic comorbidity in longitudinal studies: development and validation. *J Chronic Dis* 1987; 40: 373-383. DOI: 10.1016/0021-9681(87)90171-8.
2. Reisberg B, Ferris SH, de Leon MJ and Crook T. The Global Deterioration Scale for assessment of primary degenerative dementia. *Am J Psychiatry* 1982; 139: 1136-1139. DOI: 10.1176/ajp.139.9.1136.
3. Fried LP, Tangen CM, Walston J, et al. Frailty in older adults: evidence for a phenotype. *J Gerontol A Biol Sci Med Sci* 2001; 56: M146-156. DOI: 10.1093/gerona/56.3.m146.
4. Morisky D and DiMatteo M. Improving the measurement of self-reported medication nonadherence: Final response. *J Clin Epidemio* 2011; 64: (3):262-263.
5. Morisky D, Green L and Levine D. Concurrent and Predictive Validity of a Self-Reported Measure of Medication Adherence and Long-Term Predictive Validity of Blood Pressure Control. *Medical care* 1986.
6. Morisky DE, Malotte CK, Choi P, et al. A patient education program to improve adherence rates with antituberculosis drug regimens. *Health Educ Q* 1990; 17: 253-268.
7. Adams HP, Jr., Bendixen BH, Kappelle LJ, et al. Classification of subtype of acute ischemic stroke. Definitions for use in a multicenter clinical trial. TOAST. Trial of Org 10172 in Acute Stroke Treatment. *Stroke* 1993; 24: 35-41. DOI: 10.1161/01.str.24.1.35.
8. Stern AF. The hospital anxiety and depression scale. *Occup Med (Lond)* 2014; 64: 393-394. DOI: 10.1093/occmed/kqu024.

*MMAS-4 scale republished by permission of Adherence. © MMAS www.adherence.cc
